# Off-label and unlicensed drug use in Ayder comprehensive specialized hospital neonatal intensive care unit

**DOI:** 10.1186/s13052-020-0809-5

**Published:** 2020-04-03

**Authors:** Meles Tekie Gidey, Yohannes Gebrehaweria Gebretsadkan, Afewerki Gebremeskel Tsadik, Abraham Gebrezgabiher Welie, Brhane Teklebrhan Assefa

**Affiliations:** 1 Pharmacoepidemiology and Social pharmacy Course and Research Unit, School of Pharmacy, College of Health Sciences, Mekelle University, Mekelle, Ethiopia; 2 Department of Clinical Pharmacy School of Pharmacy, College of Health Sciences, Mekelle University, Mekelle, Ethiopia; 3 Department of Pharmacology and Toxicology, School of Pharmacy, College of Health Sciences, Mekelle University, Mekelle, Ethiopia

**Keywords:** Off-label use, Unlicensed use, Drug-use, Neonate, Intensive care unit

## Abstract

**Background:**

Off- label drug use refers to the use of medicines outside of their marketing authorization with respect to dose, dosage form, route of administration, indication or age. Off-label/unlicensed drug use significantly associated with adverse drug reactions and medication errors in neonates and critically ill neonates are more vulnerable to these problems.

**Objective:**

To assess the prevalence and associated factors with off-label and unlicensed drug use in neonatal intensive care unit of Ayder Comprehensive Specialized Hospital.

**Methods:**

A cross-sectional study was conducted from March 01,2019 to April 30, 2019 in neonatal intensive care unit of Ayder Comprehensive Specialized Hospital. Neonates admitted for 24 h and took at least one medicine were included in the study. Data was collected from prescription and medical charts. The off-label and license status of the medicine was verified based on European medicine Agency electronic medicine compendium. Data was analyzed by SPSS version 21.0. Binary and multivariate logistic regression was done to assess the predictors of off-label/unlicensed medicine use at *p-*value ≤0.05 significance level.

**Result:**

A total of 364 medicines prescribed for 122 neonates were analyzed. The prevalence of off-label and unlicensed drug use was 246 (67.58%), and 86 (23.63%) respectively. Of the total 122 neonates, 114(93.44%), and 57(46.72%) of them were exposed to at least one off-label and unlicensed drug respectively. Antibiotics were the most commonly prescribed off-label and unlicensed drugs. No statistically significant association was found between demographic as well as health related variables with off-label/unlicensed medicine use at *p-*value of ≤0.05 significance level.

**Conclusion:**

Off-label and unlicensed medicine use was high among neonates admitted to intensive care unit of the hospital. Selecting the safest medicines for such vulnerable patients is crucial to promote rational prescribing and better therapeutic benefit.

## Background

Two-thirds of the medicines used to treat pediatric patients do not have appropriate information regarding their safety and efficacy for use in these population. Pediatric patients are treated with medicines that are not tested for safety and efficacy or their uses are frequently supported by low quality of evidence for safety and efficacy [1, 2]. The situation is worse when the patients are neonates, due to their unique physiology where the pharmacokinetic evidence of medicines in older patients cannot be extrapolated [3]. Therefore, in the absence of standard prescribing information about the medicine, clinicians might be forced to prescribe medicines in unlicensed or off-label manner [2, 4].

Off- label medicine use refers to the use of medicines outside of their marketing authorization (product license) with respect to dose, dosage form, route of administration, indication or age [2, 3]. Unlicensed use refers to a medicine that does not have a marketing authorization (not authorized and licensed in a country), does not have suitable formulation in the market or extemporaneously prepared products (i.e. modified administration of licensed products) [3, 5–8]. Off-label and unlicensed medicine use is not necessarily incorrect and has to be considered when there is no other option, but it does cause risks and complications to the patient’s condition [3, 9].

Off-label and unlicensed medicine use in the newborn increases the risk of adverse drug reaction (ADR), medication errors and misuse [3, 10]. According to a literature review by Cuzollin in 2014, the risk of ADRs due to off-label/unlicensed drug use in pediatric population comprised of neonates is reported to be in the range of 23 to 60%, indicating the association of off-label/unlicensed drug use with increased risk of ADRs. Off-label/unlicensed medicine use also found to be significantly associated with increased risk of medication errors in neonatal intensive care unit (NICU), up to 8 times greater than in other departments [7].

Despite the increased risk of medication error and ADRs, the incidence of off-label/unlicensed medicine is highest among neonates admitted to NICU. For instance many studies reported off-label/unlicensed drug exposure in NICU patients in the range of 38 to 99.5% [3, 9, 11–14] and 1.9% up to 24% [3, 11, 14–16] respectively.

Little is known about the use of off-label/unlicensed medicine among NICU patients in Ethiopia. A cross-sectional study from University of Gondar reported that 75% of the prescribed medicines for pediatric patients were off-label [17]. The problem might be worst among neonates where the availability of maternal and child health medicines is very low [18] and due to the challenges of getting approved suitable medicine formulations for these patient groups. Considering the concerns of increased risk of ADRs and medication errors with off-label/unlicensed medicine use, it is imperative to assess the status of their use among NICU patients to have base line evidence. Therefore, the objective of this study was to assess the prevalence and factors associated with off-label and unlicensed drug use among neonates admitted to the NICU of Ayder Comprehensive Specialized Hospital (ACSH), a teaching hospital in Mekelle university, Mekelle, Ethiopia.

## Methods

### Description of the study design

This cross-sectional study was conducted in NICU of ACSH, Mekelle city, Northern Ethiopia. All medicines prescribed to neonates admitted to the NICU of the hospital from March 01,2019 to April 30, 2019 were assessed for their off-label and license status.

Neonates (age 0–28 days) admitted at NICU for at least 24 h and have been prescribed at least one medication to treat their medical condition, were included in the study. Neonates maintained only on oxygen therapy, parenteral nutrition, blood products, antiseptics, vaccines and intravenous fluid such as normal saline and dextrose and those having incomplete information in their medical chart/prescription were excluded from the study.

### Data collectors and data collection procedure

The data was collected from medical chart and prescriptions by a trained data collector using a structured data collection form. The data collection form includes the patients demographic details: diagnosis, NICU stay, treatment outcome and drug related data such as the number of prescribed medicine, the medicines brand/generic name, category of the medicine, dose, dosage form, route of administration, dosing frequency and indication. Neonates were categorized into three groups pre-term neonates (≤36 weeks gestational age) term neonates (37–39 weeks gestational age) and post-term neonates (≥40 weeks gestational age) [19].

The prescribed medicines were classified into three categories; off-label, unlicensed and licensed based on the European Medicine Agency electronic medicine compendium. For the purpose of this study, off-label medicine use is defined as the administration of a drug in a different manner from the recommended one in the marketing authorization with regard to age, dose, dosing frequency, administration route, or indication. Similarly licensed medicines are medicines which were prescribed and administered, following the terms of their marketing authorization. Unlicensed use refers to a medicine that does not have a marketing authorization (has no authorization license in a country), does not have suitable formulation in the market or it is extemporaneously prepared product (i.e. modified administration of licensed products).

### Statistical analysis

The collected data was checked and cleaned for consistency by an independent third party on daily basis. The data then was entered and analysed by using SPSS Version 21.0. Frequencies and percentages were calculated for all variables which were related to the objectives of the study. Binary logistic regression analysis was computed to assess the association of demographic/health related variables with off-label/unlicensed drug use at 95% CI and *p*-value ≤0.05 significance level.

### Ethical considerations

Ethical clearance was obtained from ethical review board of School of Pharmacy, College of Health Sciences, Mekelle University. The study was conducted after getting official permission from the hospital administration.

## Results

### Demographic and health related characteristics of the study population

A total of 133 neonates were admitted to the NICU during the study period and 122 of them were included into the study and the rest was excluded due to incomplete information. Of the 122 participants, male preponderance 72 (59%) was seen, and (56.6%) were term neonates. The mean age of the neonates was 1 day, majority (91%) of them being under the age of 7 days. The mean number of hospitalization stays was 6.41 ± 5.98 days (ranging from 1 to 32 days) with a total of 307 days of hospitalization for all the neonates. The neonates have been prescribed an average of 3.02 ± 1.40 medicines per prescription. The most common admission diagnosis was early onset neonatal sepsis 63(51.6%), accompanied by, respiratory distress syndrome 13 (10.7%) and late onset neonatal sepsis 6(4.9%). (Table 1 describes the demographic and health related details).

**Table 1:** Demographic and health related detail of neonates in ACSH, NICU, April 2019 (*n* = 122)

Variables	Frequency (%)
**Gender**	
Male	72 (59%)
Female	50 (41%)
**Residence**	
Rural	49 (40.2%)
Urban	73 (59.8%)
**Gestational age at birth**	
Pre-Term	45 (36.9%)
Term	69 (56.6%)
Post Term	8 (6.6%)
**Age category**	
≤ 7 days	111 (91%)
8–14	11 (9%)
Median age	1 day
**Weight (kg)**	
Mean ± SD	2.54 ± 0.79
**Hospital NICU stay(day)**	
≤ 7	95 (77.86%)
8–14	14 (11.48%)
≥ 15	13 (10.66%)
Mean ± (SD)	6.41 ± 5.98 days
**No. medicine per prescription**	
≤ 2	64 (52.5%)
3–5	50 (41%)
≥ 5	8 (6.5%)
**Admission Diagnosis**	
Early onset neonatal sepsis	63 (51.6%)
Respiratory distress syndrome	13 (10.7%)
Late onset neonatal sepsis	6 (4.9%)
Others	40 (32.8%)

### Off-label and unlicensed medicine use

**Fig. 1:**
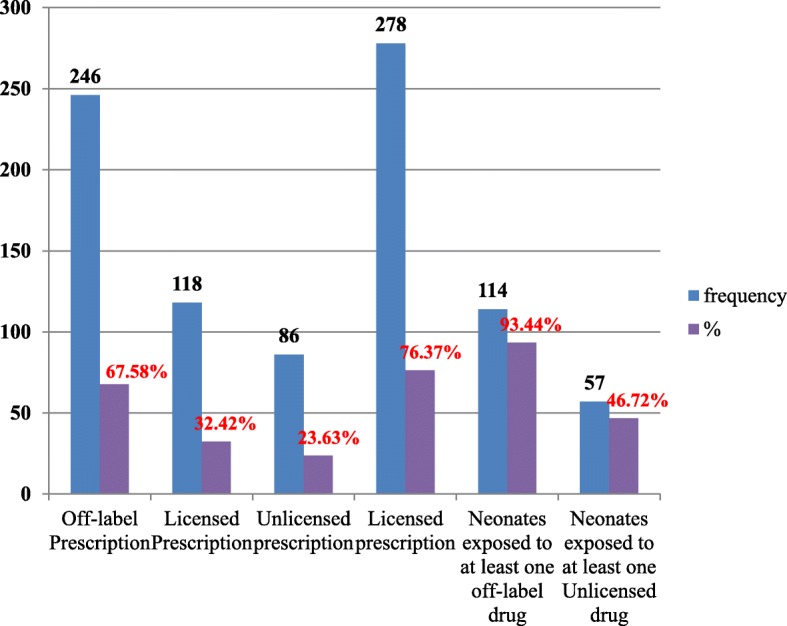
Magnitude of off-label and unlicensed drug in ACSH NICU, April 2019

From the total of 122 neonates included in the study 114 (93.44%) of them were exposed to at least one off-label medicines. In addition, 57 (46.72%) of them were exposed to at least one unlicensed medicines. Considering the total number of medicines prescribed to the neonates (*n* = 364), the prevalence of off-label and unlicensed medicines prescriptions were 246 (67.58%) and 86(23.63%) respectively (Fig. 1).

Prescriptions containing at least one off-label medicine were relatively higher among post-term (8/8) and pre-term (43/45) neonates (Table 2).

**Table 2:** Off-label and unlicensed drug use by gestational age in ACSH NICU, April 2019 (*n* = 122)

Gestational age	Frequency	Frequency(%) of neonates exposed to at least one off-label drug	Frequency(%) of neonates exposed to at least one unlicensed drug
Term	69	63(91.30%)^a^	30(43.50%)^a^
Pre-term	45	43(95.55%)^a^	22(48.90%)^a^
Post-term	8	8 (100%)^a^	5 (62.5%)^a^
**Over all**^b^	**122**	**114 (93.44%)**	**57 (46.72%)**

^**a**^The percent is calculated for each of gestational age group

^b^Indicates the overall off-label and unlicensed drug use for *n* = 122

The most widely prescribed off-label medicines categories in NICU were antibiotics (187, 51.38%) followed by Non-steroidal anti-inflammatory agent (NSAID) (17, 4.67%) and medicines prescribed for seizure (15, 4.12%). Similarly, antibiotics were also the most commonly prescribed among the unlicensed medicines (31, 8.52%) (Table 3).

**Table 3:** Off-label and unlicensed drug use by medicines category in ACSH NICU, April 2019 (*n* = 364)

Drug class	No .of prescribed medicine	Off-label use	Unlicensed use
		Frequency (%)	Frequency (%)
Antibiotics	298	187 (51.38%)	31 (8.52%)
NSAIDs	17	17 (4.67%)	17((4.67%))
Anti-epileptics	15	15 (4.12%)	15 (4.12%)
Vitamins and Minerals	12	10 (2.75%)	6 (1.66%)
Diuretics	7	7 (1.92%)	7 (1.92%)
Respiratory Drugs	7	7 (1.92%)	7 (1.92%)
Others	8	3 (0.82%)	3 (0.82%)
Total	364	246 (67.58%)	86 (23.63%)

The most commonly prescribed off-label medicine was ampicillin 99(27.2%) followed by Vancomycin 23(6.33%) and Ceftazidim 21(91.3%). Lack of neonatal use information’s, and higher dose than the recommended were the main reasons for the off-label use. Paracetamol, phenobarbital, and aminophylline were the most commonly used unlicensed medicines. Similarly, lack of information regarding neonatal use and inappropriate dosage form were the reasons for unlicensed use (Table 4).

**Table 4:** Most common off-label and unlicensed medicines prescribed in ACSH NICU, April 2019

Drug	Total medicines prescribed	Frequency(%) of off-label use	Reason for off-label use	Frequency(%) of unlicensed use	Reason for unlicensed use
Ampicillin	113	99 (27.2%)	Dose too high		
Vancomycin	23	23 (6.32%)	Dose too high	23 (6.3%)	Lack of suitable dosage form
Ceftazidim	23	21 (5.77%)	Dose too high and in appropriate frequency		
Ceftriaxone	18	16 (4.4%)	Dose too high		
Paracetamol suppository	14	14 (3.85%)	contraindicated for neonatal use	14 (3.8%)	Unauthorized for neonatal use
Cefotaxime	17	13 (3.57%)	Dose too high		
Phenobarbital Tablet	10	10 (2.75%)	Unauthorized dosage form for age	10 (2.8%)	Lack of suitable dosage form
Aminophylline injection	7	7 (1.92%)	contraindicated for neonatal use	7 (1.9%)	Unauthorized for neonatal use
Vitamin K	6	5 (1.38%)	Dose too high		
Furosemide Tablet	5	5 (1.37%)	Dose too high	5 (1.4%)	Lack of suitable dosage form
Gentamycin	84	5 (1.37%)	Dose too high		
Others	44	28 (7.69%)	Different reason	27 (7.4%)	Different reason
Total	364	246 (67.58%)		86 (23.6%)	

On binary logistic regression analysis there was no statistically significant association between demographic/health related variables and off-label/unlicensed medicine use at *p-*value < 0.05 significance level.

## Discussion

This study evaluated 364 medicines prescribed for 122 neonates for their off-label and license/authorization status.

Accordingly, the overall off-label medicine use in ACSH NICU was found to be 67.58%. This was higher compared to several study findings elsewhere in the globe which reported off-label medicine use in the range of 23 to 62% [3, 4, 9, 11, 13, 14, 20–23]. However, it was slightly lower compared to a study from India which reported an off-label prescription of 70% [2]. The differences might be attributed by the differences in availability of medicines approved for neonatal use, study design, samples size or differences pediatric medicine use policy.

In this study, high number of neonates were found to be exposed to off-label and unlicensed medicines. Of the total number of neonates included in this study 93.4% of them were exposed to at least one off-label medicine prescription. This is slightly higher compared to studies conducted in Germany, Ireland and Iran which reported 69.7–89.9% [3, 9, 11]. But, this was slightly lower compared to other studies which reported 95.5–99.5% exposure to off-label medicines [12–14]. This can be due to lack of evidence on safety and efficacy of the medicines for neonatal use due to their limited data from premarketing studies. As a result the health care providers might be forced to prescribe off-label medicines due to lack of options.

Regarding unlicensed medicine use, 46.7% of the neonates were exposed to at least one unlicensed medicine. Of the total (364) prescribed medicines only 23.63% of them were unlicensed for neonatal use. This is higher compared to reports of many studies conducted elsewhere which reported unlicensed medicine use in the range of 1.9–19% [3, 11, 13, 22, 23]. However, lower compared to a study done in India in which 56% of the prescriptions were unlicensed for neonatal use [4]. The difference might be related to the differences in medicines licensing policy across the countries, disease pattern difference or differences in definitions of unlicensed medicines used in the other studies.

The use of off-label and unlicensed medicines was found to differ by the gestational age of the neonates. Many studies elsewhere in the world reported high rate of off-label [11, 12, 22, 24] and unlicensed [11] medicine use among pre-term neonates. Despite the number of post-term neonate were very small in our study, off-label prescriptions were relatively higher for post-term and preterm neonates where (8/8) and (43/45) of the post-term and pre-term neonates were exposed to at least one off-label medicines respectively. Similarly post-term and pre-term neonates were also found to be exposed to unlicensed medicines at higher rate where (5, 62.5%) and (22, 48.9%) of the post-term and pre-term neonates were exposed to at least one unlicensed medicines respectively. The high number of off-label and unlicensed medicine use in post-term and preterm neonates in the present study might be related with scarcity of medicines that are suitable for use in the mentioned neonatal categories.

In the present study, antibiotics were the most commonly used off-label medicines compared to the other classes of medicines. This was similar finding with that of study reports from Iran and Italy [3, 24]. However, it was different from a study conducted in Netherlands which reported that blood products were the top class of medicines used in an off-label manner [20]. The difference might be related with difference in disease status. On the other hand, antibiotics were also found to be the most commonly prescribed medicines in unlicensed manner in our study. This was in consistence with a study finding from Iran [3]. But studies from southern Italy and Irish neonatology reported caffeine as the most commonly used medicine in an off-label manner. This can be explained by differences in disease epidemiology across the study settings.

The most commonly prescribed off-label medicine was ampicillin 99(27.2%) followed by Vancomycin 23(6.33%) and Ceftazidim 21(5.77%). The main reason for the off-label use of medicines were lack of neonatal use information’s, and dose higher than the recommended. The same reason was also reported from a study conducted in Iran [3]. However our finding were different from studies conducted in Germany, Brazil and India which reported the commonest reason for being off-label as frequency of administration, dosage form difference, and dose respectively [4, 9, 13]. This is expected since such difference might be atributed to differences in medicines licensing policy across the countries, disease pattern difference or difference in definitions of off-label medicines used. Paracetamol, phenobarbital, and aminophylline were the most commonly used unlicensed medicines due to their lack of neonatal use information and inappropriate dosage forms for neonatl patients. The same resason was cited from a study done in India [4].

As a limitation, the study was conducted in small number of study participants for short period of time in a single center; thus further study is needed to strengthen the generalizability of this evidence. In addition, the data was collected from patients’ medical records and prescriptions; therefore, reason for off-label and unlicensed use and the possible harmful side effects of this off-label/unlicensed use were beyond the scope of this study.

## Conclusion

Off-label and unlicensed medicine prescriptions are very common among neonates admitted to intensive care unit of ACSH. Selecting and identifying the safest medicines for these vulnerable patients is necessary to promote rational prescribing and better therapeutic benefit.

## Data Availability

All datasets from which we derived our conclusion is deposited in SPSS software and it can be accessed from the corresponding author on reasonable request.

## Abbreviations

ACSH: Ayder Comprehensive Specialized Hospital
ADR: Adverse Drug Reaction
NICU: Intensive Care Unit
